# The 2,240-Atom Unit Cell of PrMg_1.6_Zn_5.4_: An Intergrowth of the Laves and Heusler Structures Illustrating a Mechanism for the Stabilization of Complex Intermetallics

**DOI:** 10.1021/jacs.5c12386

**Published:** 2025-09-16

**Authors:** Danica G. Gressel, Leah C. Garman, Daniel C. Fredrickson

**Affiliations:** Department of Chemistry, 5228 University of Wisconsin-Madison, 1101 University Avenue, Madison, Wisconsin 53706, United States

## Abstract

The crystal structures of intermetallic phases encompass a fundamental mystery about the chemistry of metals: what drives atoms that prefer simple sphere packings as pure elements to adopt complex structures, sometimes with unit cells containing thousands of atoms? We recently developed the interface nucleus concept to interpret their most complicated arrangements in terms of chemical pressure (CP)-driven assemblies of modules of simple parent structures. The predictions of this approach earlier led us to the Pr–Mg–Zn system and the discovery of the modular structure of PrMg_4_Zn_10_ built from MgZn_2_- and EuMg_5_-type units. Herein, we present the structure of a second intergrowth compound in this system that exhibits far greater complexity: PrMg_1.6_Zn_5.4_, whose 34.50 Å cubic unit cell contains 2,240 atoms. Its structure can be simply decoded in terms of a 5 × 5 × 5 supercell of the Heusler structure (or alternatively a 10 × 10 × 10 supercell of the body-centered cubic (BCC), structure) embedded with Laves phase fragments. The nearly seamless transition between the Heusler and Laves domains observed here reflects a simple relationship between these structures, in which they can be interconverted by the exchange of a tetrahedron of atoms for a single larger atom (MgCu_2_ type to BCC), or vice versa (BCC to MgCu_2_ type). Driving forces underlying PrMg_1.6_Zn_5.4_’s structure are inferred from the CP analysis of the parent structures, which highlight the overcompression of the Pr atoms in a model Heusler phase and the opportunities for relief by expanded environments available at the boundaries between the Laves and Heusler domains in PrMg_1.6_Zn_5.4_.

## Graphical abstract

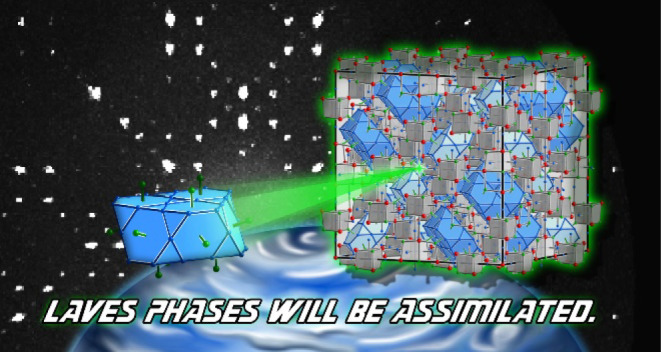

## 1 Introduction

Linus Pauling’s report on investigations into the NaCd_2_ structure in 1923 provided an early glimpse into the potential for incredible complexity to emerge in intermetallic phases.^1^ The contents of its 30.56 Å cubic unit cell were eventually outlined by Samson nearly 40 years later,^2^ who derived an approximately 1,152-atom arrangement from geometrical and symmetry-based reasoning. Since then, intermetallics with similar or greater complexity have been discovered, with giant unit cells containing thousands of atoms, the atomic-level details of which, in some cases, remain unresolved.^3,4^

How does such complexity arise among metallic atoms, which we assume would prefer simple sphere packings? The crystal structures themselves seem to suggest that there are several distinct answers to this question. The large unit cell of Eu_4_Cd_25_ (*cF*1,392)^5,6^ can be rationalized from its close relationship to Tsai-type quasicrystals,^7,8^ which are aperiodic (though long-range ordered) in 3D space.^9^ The Al_56.6_Cu_3.9_Ta_39.5_ (*cF*5,908) and Al_55.4_Cu_5.4_Ta_39.1_ (*cF*23,134)^10,11^ structures involve the intergrowth of Laves-phase and fullerene-like cage domains. NaCd_2_ (*cF∼*1,152), β-Al_3_Mg_2_ (*cF∼*1,227),^12^ Cu_4_Cd_3_ (*cF∼*1,124),^13^ and YbCu_4.5_ (*mC*7,448),^3,4^ on the other hand, are built from nanometer-scale blocks of simpler structures. Additionally, the ordered vacancy patterning of Lu_37_Ru_16.4_In_4_ (*cI*918) creates an 8 × 8 × 8 BCC superstructure.^14^ Finally, the giant cubic Tb_117_Fe_52_Ge_112_ type (*cF*∼1124)^15–20^ is based on a hierarchical construction in which the atoms of a simple structure are replaced with multishelled clusters.^21^

In most of these cases, mystery still surrounds the driving forces leading to these geometrical themes and how those forces might be leveraged to influence the properties of a material. In this article, we present the discovery of a new member of this class of extremely complicated intermetallics, PrMg_1.6_Zn_5.4_, whose 2,240-atom unit cell points to a surprisingly simple cause for the intergrowth of two parent structures.

Our initial interest in the Pr–Mg–Zn system was sparked by predictions made in the context of the Interface Nucleus Approach^22–24^ to understanding intermetallic structures with modular architectures. Within this model, interfaces between domains of two parent structures are facilitated by shared geometrical motifs, which serve as docking points. Complementarity in the packing stresses at these motifs in the two structures, as analyzed with the DFT-Chemical Pressure analysis,^25–28^ provides an impetus for the formation of these interfaces. With this approach, the compatibility of potential intergrowth partners sharing a specific motif can be assessed as a guide for new syntheses. Our first iteration of this approach highlighted the combination of the EuMg_5_ ^29,30^ and MgCu_2_ ^31^ or MgZn_2_ ^32^ types as having strong potential, immediately suggesting the synthetic exploration of lanthanide–Mg–Zn and lanthanide–Mg–Cu systems.

Among these systems is Pr–Mg–Zn, previous studies of which indicated the existence of four phases for which no structure had been determined.^33,34^ Synthesis in this system quickly yielded crystals of PrMg_4_Zn_10_, which are built from two of the expected parent structures, the EuMg_5_ and MgZn_2_ types. The phase diagram, however, indicates that additional structural chemistry remains to be discovered. Here, we describe a second intergrowth structure that we have since encountered: PrMg_1.6_Zn_5.4_.

As we will see below, its complex structure can be understood as a host–guest arrangement involving a matrix based on the Heusler structures that encloses units of a Laves phase. As their historical names imply, these are two of the most common intermetallic structures, which separately form their own fields of study within materials science, each with its own superstructure chemistries.^35–38^ The Laves phases, which encompass the cubic MgCu_2_ type, the hexagonal MgZn_2_ and MgNi_2_ types,^39^ and a variety of long-period variants,^40–42^ boast more than 1,300 entries in the Inorganic Crystal Structure Database (ICSD).^43^ They are notable for applications as hydrogen storage materials and electrodes in metal-hydride batteries,^44–46^ magnetocaloric materials,^47,48^ superconductors,^49^ and corrosion- and oxidation-resistant phases.^50^ Heusler compounds^51^ meanwhile, are ordered variants of the BCC structure (with a face-centered supercell) that represent more than 1,000 entries in the ICSD and are pursued as shape memory alloys,^52^ superconductors,^53,54^ and magnetocaloric materials.^55,56^

The structure of PrMg_1.6_Zn_5.4_ offers the prospect of hybrid materials combining the functionality of these two classes of intermetallics. As such, understanding the factors stabilizing its modular arrangement over the separated parent structures has become a pressing issue. Using DFT-Chemical Pressure (CP) analysis, we observe that the favorability of a Pr–Mg–Zn Heusler structure is limited by the large atomic size of the Pr atoms. However, a close structural relationship between the Heusler and MgCu_2_-type structures provides a solution, with the boundaries between epitaxially matched domains of the two types offering expanded coordination environments to the Pr atoms. In fact, every Pr atom in PrMg_1.6_Zn_5.4_ is positioned at the domain interfaces. In this way, the formation of complex intermetallics is connected to the phenomenon of elemental segregation at grain boundaries,^57^ an effect noted in lanthanide-magnesium alloys.^58^

## 2 Experimental Section

### 2.1 Synthesis of PrMg_1.6_Zn_5.4_

Samples were prepared using elemental Pr filings (from a piece of Pr metal procured from Ames Laboratory, 99.99%), Mg chips (Aldrich, 99.8%, −4 + 30 mesh), and Zn powder (Alfa Aesar, 99.9%, −100 mesh). The elements were weighed in a Pr:Mg:Zn molar ratio of 1:6:3 to form ∼0.5 g samples. The starting materials were ground to maximize homogeneity using a mortar and pestle, pressed into pellets, loaded into alumina crucibles (fashioned from an alumina crucible by adding a cement base), capped with a second crucible, and sealed under vacuum in fused silica tubes. The samples were brought to 700 °C, where they were held for 12 h and then cooled to 540 °C. After 24 h at this temperature, the samples were quenched in ice water. The synthesis resulted in gray truncated triangular crystals suitable for single-crystal X-ray diffraction analysis. Following the determination of the PrMg_1.6_Zn_5.4_ structure, subsequent samples were prepared according to the molar ratios derived from single-crystal data. These samples were annealed at 540 °C for 10 days and quenched in an ice water bath.

### 2.2 Powder X-ray Diffraction

Representative pieces of the samples were ground into a powder using an agate mortar and pestle and mounted onto a zero-diffraction Si background plate. Measurements were taken with a Bruker D8 Advance Powder X-ray Diffractometer using Cu Kα radiation (λ = 1.5418 Å) over a 2θ range of 10–90° in 0.02° increments with an exposure time of 0.9 s. These powder patterns were analyzed using *Match!* software.^59^

### 2.3 Single-Crystal X-ray Diffraction

Gray crystals with truncated triangular shapes were picked from the sample and secured to thin glass fibers with epoxy. Data sets were collected on a Bruker Quazar SMART APEX2 diffractometer with a Mo Kα (λ = 0.71073 Å) IμS source. The APEX3 ^60^ software was used to process run lists and frame data. The diffraction patterns of the single crystal were indexed to a cubic cell with *a* = 34.50 Å. Inspection of the Laue symmetry and systematic absences in the reconstructions of the reciprocal lattice layers indicated *Fd*3̅*m* (No. 227) as the highest possible space group assignment. The charge-flipping algorithm in the *SUPERFLIP* ^61^ program within the *JANA2006* ^62^ software agreed with the space group assignment and found 29 symmetry-distinct atomic positions. Structure refinement was performed on *F* ^2^ in *JANA2006,* giving a structure with a refined composition of PrMg_1.593(2)_Zn_5.407(2)_. The structure was refined with 2 mixed Mg/Zn sites, each with their overall occupancy set equal to 1.

### 2.4 Elemental Analysis via Wavelength-Dispersive X-ray Spectroscopy (WDS)

The sample with high phase purity was selected for elemental analysis. Small pieces of the sample were embedded in epoxy at the bottom of a segment of aluminum tubing. Once the epoxy had cured, a smooth surface exposing the sample pieces was created by polishing with diamond lapping films (grits 9–0.5 μm), and the sample was carbon-coated. WDS measurements were performed with a Cameca SX100/SX-Five electron microprobe using standards PrF_3_-MAC, Mg metal, Zn metal, and ZnO for the Pr *L*_α_, Mg *K*_α_, Zn *K*_α_, and O *K*_α_ transitions, respectively. Oxygen was included in case oxidation of the sample had occurred after polishing, but no quantifiable oxygen content was found in the sample. Measurements were taken with a beam energy of 20 keV and a current of 20 nA. Scanning electron microscope (SEM) backscattered electron (BSE) images and WDS data for individual points are given in the Supporting Information.

### 2.5 Magnetic Properties Measurements

DC magnetization measurements were taken using a Quantum Design MPMS3 Superconducting Quantum Interference Device (SQUID) magnetometer. A powdered portion of the sample was loaded into a gel capsule, which was inserted into a straw. Zero-field-cooled (ZFC) and field-cooled (FC) magnetization measurements were collected at 10 and 1,000 Oe over the temperature range of 1.8–400 K. Magnetic field-dependent hysteresis curves were also collected at temperatures of 1.8, 2, 15, 50, 100, and 300 K over a range of 5000 to −5,000 Oe. Data were corrected for the sample holder (for the magnetic susceptibility measurements) and sample diamagnetism^63^ then plotted using an in-house MATLAB program. The high-temperature susceptibility can be attributed to the Curie–Weiss behavior arising from Pr^3+^ moments. Below ∼50 K, very weak features arise that may be due to ferrimagnetism, but the role of ferromagnetic impurities cannot be excluded. The corresponding data and further details are presented in the Supporting Information.

### 2.6 DFT-Chemical Pressure (CP) Analysis

DFT-CP schemes for MgZn_2_ and an ordered model of the Heusler compound YMg_1.5_Zn_1.5_ ^64,65^ were analyzed to explore how atomic-packing issues in these parent structures relate to the formation of PrMg_1.6_Zn_5.4_. CP data for MgZn_2_ were obtained from the Intermetallic Reactivity Database,^66,67^ while that for YMg_1.5_Zn_1.5_ was newly generated for this work. An ordered model with the composition YMgZn_2_ was first geometrically optimized with the *ABINIT* software^68–72^ using LDA-DFT^73^ and Hartwigsen–Goedecker–Hutter norm-conserving pseudopotentials.^74^ Single-point calculations were then carried out on the ground-state geometry, along with slightly expanded and contracted versions (linear scale 1.000 ± 0.005), to obtain the electron densities, wavefunctions, kinetic energy densities, and Kohn–Sham potential components needed for the generation of the CP schemes. Throughout these calculations, the energy cutoff was set to 125 Ha, while a Γ-centered 6 × 6 × 6 k-point mesh was employed.

The CP map for YMgZn_2_ was generated with *CPpackage3*,^75^ using the core-unwarping procedure,^76^ the mapping of nonlocal energy terms,^27^ and the self-consistent partitioning of the *E*_Ewald_+*E*_α_ contributions into localized and itinerant components.^27,28^ The free-ion densities used in the core-unwarping and contact volume construction were created with the *Ab Initio Pseudopotentials Engine (APE)*,^77^ with ion charges chosen for self-consistency within the binary iterative Hirshfeld scheme.^28^ The pressures within the contact volumes were averaged and then projected onto atom-centered spherical harmonics for visualization with the in-house *FigureTool2* program.

### 2.7 Domain Analysis with the *GrowDomain* Program

The atomic positions of PrMg_1.6_Zn_5.4_ were analyzed in terms of BCC- and MgZn_2_-type domains using the *GrowDomain* program.^78^ First, seed units for the matching process were selected from PrMg_1.6_Zn_5.4_ for the two domains: a rhombic dodecahedron from the Heusler domain and various assemblies of Friauf polyhedra for the Laves phase regions. The *ToposPro* program^79^ was used to generate graph representations of these units, their structural contexts, and their counterparts in the parent structures.

For each seed, the *GrowDomain* program reads the graph from PrMg_1.6_Zn_5.4_ and its analogue in the corresponding parent structure, determines their isomorphism, refines a transformation matrix between their coordinates, and seeks atoms in the vicinities of the seed in the two structures that are similarly mapped to each other with the same transformation matrix. The identified atoms are added to the collection of shared atoms, and the process of refining the transformation matrix and seeking new matching atoms is repeated until no further matches are found. With the transformation matrix in place, the displacement of the atoms at the edges of the domain from their idealized positions can be correlated with the CP features of the parent structure.

## 3 Results and Discussion

### 3.1 Synthetic Results

Encouraged by the modular features of the PrMg_4_Zn_10_ structure,^24^ we set out to synthetically investigate indications in the Pr–Mg–Zn phase diagram of several other compounds with unknown structures.^33,34^ A sample with a loading composition of Pr:Mg:Zn = 1:6:3 was prepared to explore phases with higher magnesium contents. The sample was heated over 8 h to 700 °C, held there for 12 h, cooled to and held at 540 °C for 1 day, and quenched in ice water. Upon opening the sample, signs of oxidation quickly appeared, as might be expected from the presence of Mg and Pr metal detected in the powder diffraction patterns. Despite the overall air sensitivity of the sample, however, crystals could be harvested from it, whose single-crystal X-ray diffraction patterns point to a cubic cell with a large *a* parameter of 34.50 Å. Our preliminary structure solution indicated a new compound with the approximate composition PrMg_1.6_Zn_5.4_.

We then pursued a high-yield synthesis of the compound using the 1:1.5:5.3 ratio from the structure solution and adjusted our heating profile to hold the samples at 540 °C for 10 days and then quench them in ice water. The resulting samples were uniform in appearance and stable in air over several months. Their powder X-ray diffraction patterns (Figure 1) show numerous sharp and largely well-resolved peaks spanning across 2θ from 13° to above 80°. All peaks show close alignment in position and relative intensity to those simulated from the refined structure of PrMg_1.593(2)_Zn_5.407(2)_.

**Figure 1:**
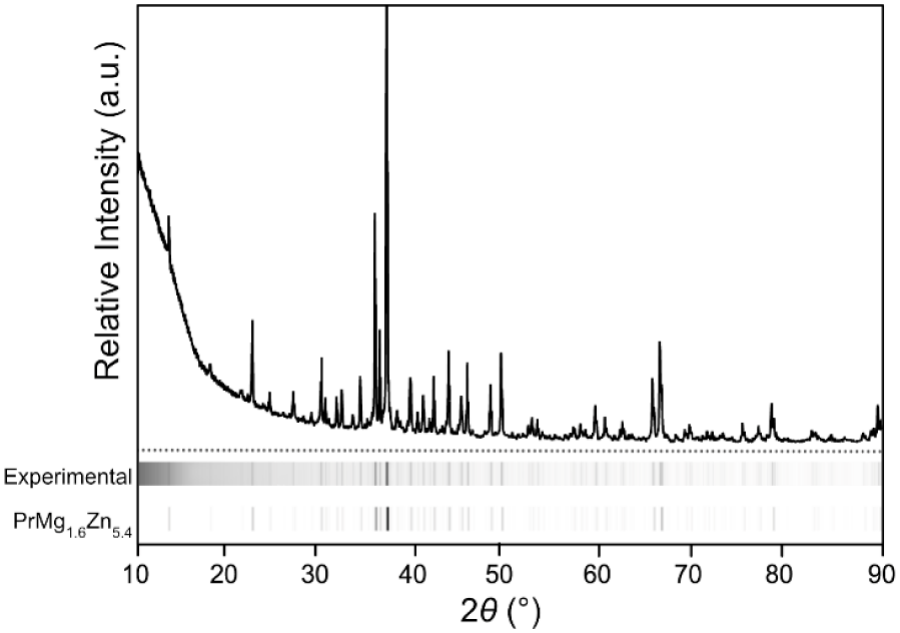
Powder X-ray diffraction pattern of a sample of PrMg_1.6_Zn_5.4_. Film-strip-style representations of the experimental data and the simulated pattern derived from the structure refined from single-crystal X-ray diffraction data are given below.

The conclusion that PrMg_1.6_Zn_5.4_ has been obtained in relatively high purity is affirmed by scanning electron microscopy-backscattered electron (SEM-BSE) imaging, which shows that a single composition dominates the sample, with the minor presence of a secondary phase. Wavelength-dispersive X-ray spectroscopy (WDS) measurements yield compositions of Pr_1.00(7)_Mg_1.954(15)_Zn_5.165(14)_ and Pr_1.00(8)_Mg_1.44(4)_Zn_3.04(18)_ for these two phases, respectively. The major phase here appears to be significantly Mg-rich relative to the refined composition, but it should be noted that quantification is difficult due to Pr and Zn being strong absorbers of X-rays emitted by Mg.

Magnetic susceptibility measurements on this sample of PrMg_1.6_Zn_5.4_ show paramagnetic behavior, with weak transitions evident at lower temperatures that may stem from ferrimagnetism or ferromagnetic impurities (see the Supporting Information).

### 3.2 Structure Solution and Refinement

Inspection of the samples under a stereo microscope revealed small, shiny, light-gray crystals shaped like truncated triangles, which were isolated for single-crystal X-ray diffraction analysis. The resulting diffraction patterns were indexed to a cubic cell of 34.50 Å, with systematic absences consistent with the space group *Fd*3̅*m* (Table 1). With this choice of symmetry, the structure solution using the charge-flipping algorithm converged on a nearly complete structure with 29 symmetry-distinct atomic positions.

**Table 1:** Crystal Data for PrMg_1.6_Zn_5.4_

Refined Composition	PrMg_1.593(2)_Zn_5.407(2)_
WDS Composition	Pr_1.00(7)_Mg_1.954(15)_Zn_5.165(14)_
Crystal Dimension (mm^3^)	0.069 × 0.0794 × 0.101
Crystal Color	Gray
Data Collection Temperature	Ambient
Radiation Source, λ (Å)	Mo Kα, 0.71073
Absorption Correction	Analytical with refined absorption coefficient
Space Group	*Fd*3̅*m* (No. 227)
*a* (Å)	34.4965(9)
Cell volume (Å^3^)	41,051.1(19)
*Z*	280
Absorption Coefficient (mm^–1^)	29.9
θ_min_, θ_max_	1.02, 30.53
Refinement Method	F^2^
R_int_ (obs., all)	0.1212, 0.1622
Number of Reflections	197796
Number of Parameters	145
Unique Reflections (I > 3σ, all)	1757, 2968
R(I > 3σ), R_w_(I > 3σ)	0.0255, 0.0564
R(all), R_w_(all)	0.0629, 0.0678
S(I > 3σ), S(all)	1.21, 1.09
Δρ_max_, Δρ_min_ (e^–^/Å^3^	3.11, −1.48

Over the course of subsequent structural refinements, three sites showed signs of potential fractional occupancy. First, the atomic displacement parameters for the Zn17 and Zn18 sites were unusually large, while the Fourier difference map suggested that the electron density on these sites was too high. They were thus modeled as mixed Zn/Mg positions, with the site occupation factors for the Zn17/Mg7 and Zn18/Mg8 sites refining to, respectively, 0.81/0.19 and 0.51/0.49. After modeling the two mixed Mg/Zn sites, the s minimum and maximum Fourier difference densities for the structure dropped from −5.17 and 9.35 electrons/Å^3^ to −1.48 and 3.11 electrons/Å^3^.

At this point, it was noted that the Mg3 site has a significantly larger *U*_equiv_ value than any of the other Mg positions, suggesting that it may be only partially occupied. However, refinement of the occupation did not yield a significant reduction, even when the refinement of the Mg3 occupation and ADPs was carefully alternated to avoid correlations. Instead, the larger ADPs for this position can be understood from its structural context: they are coordinated by a cube formed from the Zn17/Mg7 and Zn18/Mg8 sites. The positions of the Mg atoms within this cube are evidently sensitive to the local Zn/Mg occupation pattern.

### 3.3 The Crystal Structure of PrMg_1.6_Zn_5.4_

The refined structure of PrMg_1.6_Zn_5.4_ contains 2,240 atoms in its *F*-centered unit cell. When confronted with such complexity, the task of discerning the chemical meaning may seem daunting. However, upon closer inspection, the structure has a straightforward construction. Indications of simpler structural motifs become clear as we focus on the Mg sites in the structure. For example, the Mg5 atoms trace out 6-fold rings, resembling the carbon framework of chair cyclohexane (Figure 2a). Each of these Mg5 atoms sits in a Friauf polyhedron built from a truncated tetrahedron of Zn atoms capped on its hexagonal faces by Mg atoms (the Mg1 and Mg2 sites). Together, the Mg5 sites and surrounding polyhedra create a slab of the Laves phase structures, as in the MgCu_2_ or MgZn_2_ types. This Laves phase fragment accounts for 3 of the 6 symmetry-distinct Mg positions in the structure.

**Figure 2:**
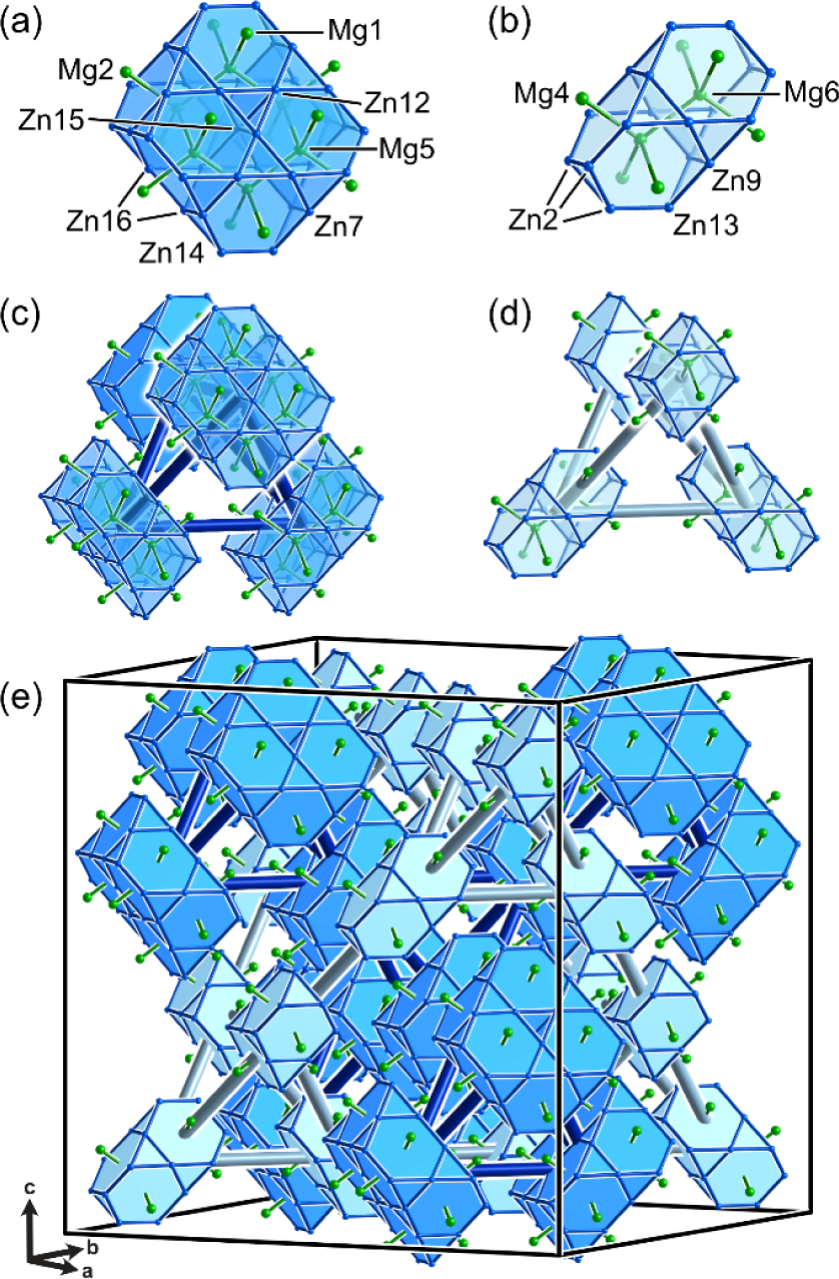
Laves phase units in the PrMg_1.6_Zn_5.4_ structure: (a) slab of six Mg5-centered Friauf polyhedra and (b) pair of Mg6-centered polyhedra forming fragments of a Laves phase; (c)-(d) tetrahedral arrangements made by the two Laves units in the structure; (e) networks created through the vertex sharing of these tetrahedral assemblies.

A second Laves phase fragment can be found around the Mg6 sites. The Mg6 atoms form dumbbells and are similarly coordinated by interpenetrating Friauf polyhedra (Figure 2b), with the Mg4 sites capping the hexagonal faces of Zn_12_ truncated tetrahedra. This unit can also be derived from the Laves phase structures.

A look at how these units are distributed throughout the unit cell gives an immediate sense of how the structure is organized. Each of the Laves phase fragments is grouped into larger tetrahedral motifs (Figure 2c,d). At a larger length scale, these tetrahedra share corners to form two interpenetrating diamond networks (Figure 2e) that thread through the entire unit cell. This description encompasses 1,296 atoms out of the 2,240 total in the unit cell, including all but one of the Mg sites (the exception being Mg3). Notably, however, none of the Pr sites are captured here, making the Laves phase motifs entirely Mg–Zn domains.

So, what about the remaining atoms in the unit cell? Clues here are found in the coordination environment of the one Mg site for which we have not yet accounted, Mg3 (Figure 3a). Its closest neighbors form a cube made of Zn17/Mg7 and Zn18/Mg8 mixed sites (designated hereafter as Zn17 and Zn18 for simplicity). The faces of the Mg3-centered cube are capped by Pr2 atoms, beginning the formation of a BCC-like framework. Building further out from the central Mg3 adds an additional 12 Mg1 atoms and completes somewhat distorted cubes of alternating Mg and Pr atoms around the Zn17 positions. Altogether, the pattern resembles a larger section of the BCC structure with a coloring pattern reminiscent of the Heusler type. In fact, almost a full unit cell of the Heusler structure is thus obtained, except that the Pr atoms needed to complete the Mg/Pr cubes around the Zn18 site are missing. In their place, tetrahedra of Zn atoms derived from the Laves phase fragments are present, seemingly signaling the end of the BCC-like connectivity.

**Figure 3:**
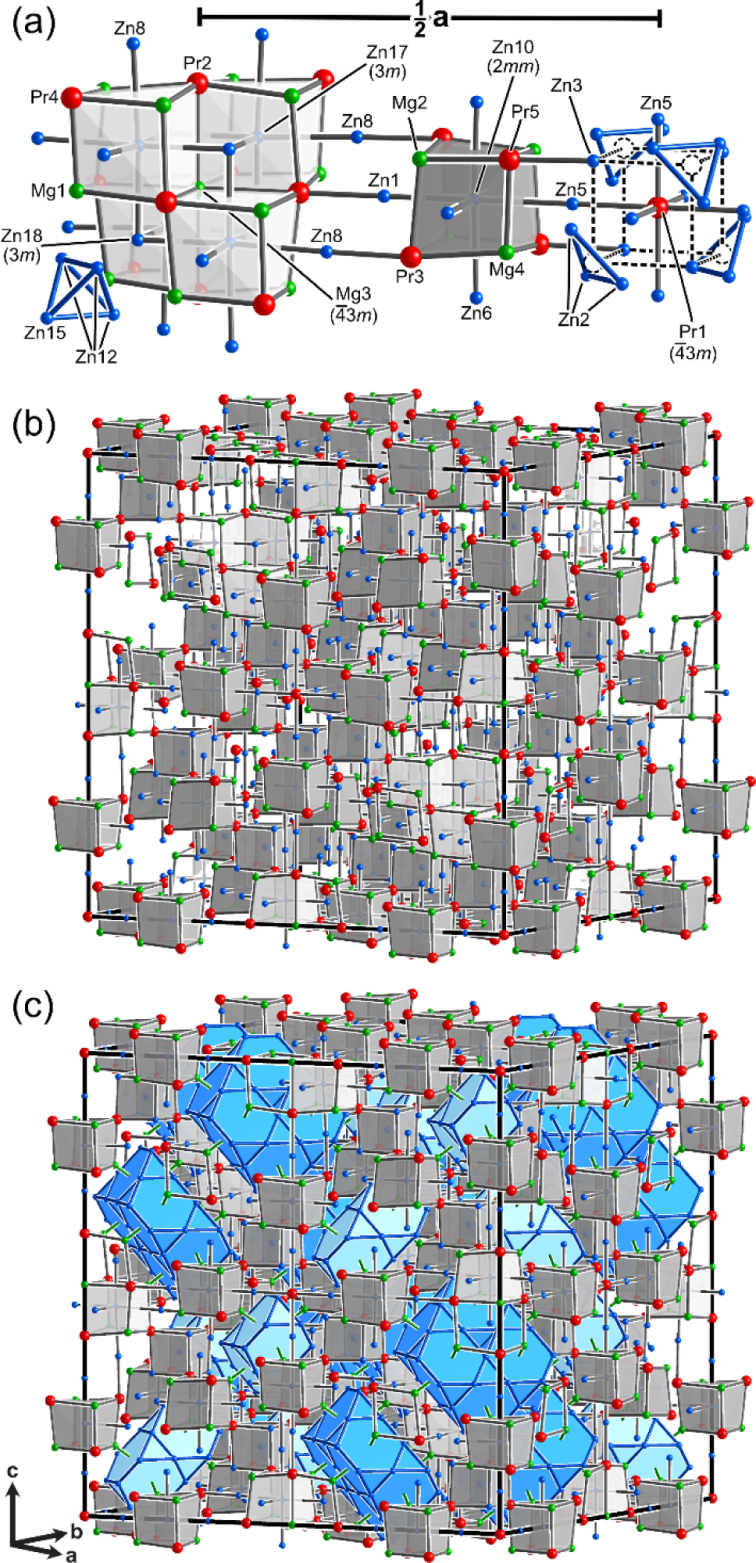
The full PrMg_1.6_Zn_5.4_ structure as a host–guest structure based on a Heusler matrix and Laves phase inclusions: (a) elements of the BCC/Heusler structure in the geometrical features around the Mg3, Zn10, and Pr1 sites; (b) propagation of this framework through the entire unit cell; (c) completion of the structure by adding the Laves phase units highlighted in Figure 2.

However, along the *a*, *b*, and *c* directions, the primitive cubic network initiated by the Zn17/Mg7 and Zn18/Mg8 sites can be propagated through the Zn1 and Zn8 sites to another cube centered on Zn10. From there, traces of the BCC pattern can be followed to the Pr1 site halfway across the unit cell from Mg3. At this point, the surroundings of the Mg3- and Pr1-centered regions merge into a single BCC/Heusler domain that spans the entire structure (Figure 3b).

In this way, the full crystal structure of PrMg_1.6_Zn_5.4_ (Figure 3c) is resolved into two basic components: a Heusler-based host matrix that runs continuously throughout the crystal and Laves phase inclusions, containing between two and six Friauf polyhedra. The large size of the unit cell then derives from the patterns formed by the Laves phase units. Indeed, based on their Wyckoff positions, the distance between the Laves unit centers is a simple function of the *a*-cell parameter: (√2)*a*/4 = 12.19 Å.

Within this scheme, the positions of the Pr atoms offer clues about the origins of the structure. Consider the Pr1 site (Figure 3a, right). It is surrounded entirely by 22 Zn atoms, which adopt a configuration that, at first glance, appears unusual. However, the Heusler/BCC structure provides a cipher for interpreting it. The Zn3 and Zn2 sites form single atoms and triangles (derived from the Laves phase units), respectively, positioned on the corners of a cube around the Pr1 atom (Figure 4). Replacing each Zn2 triangle with an atom at its center (dotted circle) would create a correspondence with the Heusler structure. Looking a bit further out, we spot Zn5 atoms capping each square face of this cube-like arrangement, continuing the BCC network. As in the Mg3-centered BCC unit, the occurrence of a Zn cluster replacing the corner atom of a cube disrupts the propagation of a BCC-like network.

**Figure 4:**
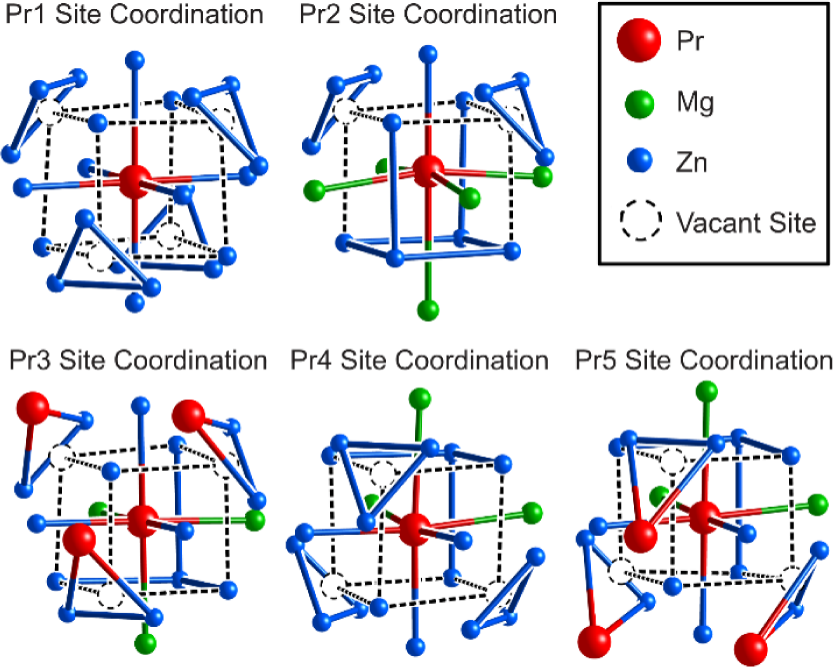
Coordination environments of the symmetry-independent Pr sites in PrMg_1.6_Zn_5.4_. Black dashed lines trace out cubes surrounding the Pr atoms to highlight the relationship to the BCC/Heusler structure. Dashed circles represent points where atoms are expected based on this relationship but are replaced by triangles.

As shown in Figure 4, the coordination environments of the other symmetry-distinct Pr sites bear similarities to those of Pr1. In each case, the atom’s surroundings can be derived from a BCC-like framework, with a selection of atoms in the cube of nearest neighbors being replaced by triangles from the Laves phase units. For Pr1, four of the eight atoms in the cube are replaced in this way. The other sites, meanwhile, have a smaller degree of substitution, with three such triangles present for the Pr3, Pr4, and Pr5 sites, and only two for the Pr2 site.

These general features result from the placement of Pr atoms at the surface of the Heusler domain, such that their coordination environments are completed by atoms from the Laves units. The overall result is that the Pr atoms gain coordination numbers that would not be possible in a simple Heusler structure, suggesting that the space requirements of these atoms may play a powerful role in the emergence of this structure. We will return to this point in a later section.

### 3.4 *GrowDomain* Analysis of PrMg_1.6_Zn_5.4_’s Domain Structure

The relationship between the BCC/Heusler and Laves phase domains of PrMg_1.6_Zn_5.4_ can be further elucidated by a computer-based assignment of atoms to the domains, as enabled by the *GrowDomain* program, in ways that support our CP analysis below. Here, we begin with a fragment of the BCC/Heusler structure from PrMg_1.6_Zn_5.4_, the coordination environment of the Mg3 site, and a corresponding unit derived from the parent BCC/Heusler structure. The *GrowDomain* program loads these units and determines the isomorphism between their atoms. It then refines a transformation mapping the atoms of the unit from PrMg_1.6_Zn_5.4_ onto the BCC/Heusler structure (Figure 5). Once the two structures are thus placed in the same coordinate system, the program explores the environments of the units in the two structures (as given by large samples of the atoms surrounding them), looking for additional corresponding atoms. The newly discovered matches are added to the group of atoms shared by the two structures, and the procedure returns to the refinement of the transformation. Through repetitions of this process, we gradually explored the extent to which the Mg3-centered BCC/Heusler domain encompasses atoms of PrMg_1.6_Zn_5.4_.

**Figure 5:**
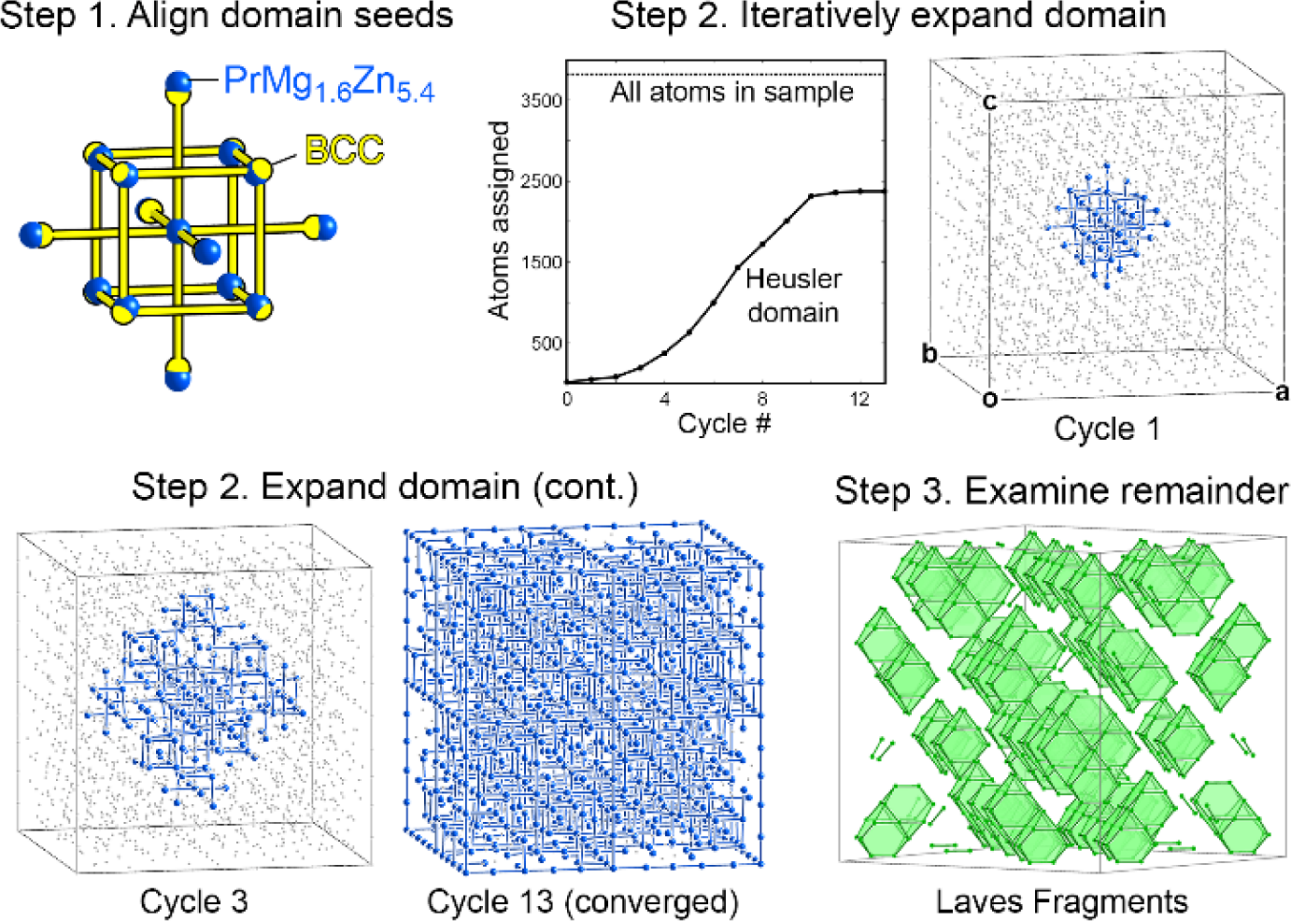
Application of the *GrowDomain* procedure to PrMg_1.6_Zn_5.4_, taking as a sample one unit cell plus the coordination environments of the atoms on the cell edges (3,817 atoms total).

As shown in Figure 5, the process in this case converges after 13 cycles, creating a Heusler domain that extends continuously through the structure along all three axes. While this result confirms our earlier conclusion, we can now ask which atoms are left over, as it cannot be assigned to the Heusler connectivity. These atoms are presented in the lower right of Figure 5; they consist solely of the atoms constituting the truncated tetrahedra of the Laves phase domains.

The Heusler/Laves phase intergrowth pattern is thus recovered, but we also learn something new. None of the Mg atoms in the Laves phase units are present among the remaining atoms. They were instead recognized by the program as extensions of the Heusler/BCC arrangement. This assignment of atoms from the Laves units to the BCC/Heusler domains hints at an epitaxial or topotactic relationship between the two structures.

### 3.5 Structural Relationships between the Parent Phases

This *GrowDomain* analysis hints at a deeper relationship between the BCC/Heusler and Laves phase structures than is evident from the usual depictions in terms of cubes and Friauf polyhedra. In this section, we will explore this connection, relating the two types through a simple substitution pattern with parallels to the features seen in the Pr coordination environments of PrMg_1.6_Zn_5.4_.

Let's consider the MgCu_2_ type, as in its cubic symmetry it seems more compatible with the BCC/Heusler structure than the other Laves phase types (Figure 6). MgCu_2_ features a diamond network of Mg atoms interpenetrated by a second diamond network of vertex-sharing Cu tetrahedra. Together, they form a pattern of interpenetrating Friauf polyhedra, in which the capping Mg atoms coming off the hexagonal faces of one truncated tetrahedron are the central atoms of the neighboring Friauf polyhedra. A comparison of the Mg environments with those of the Pr sites in PrMg_1.6_Zn_5.4_, though, offers another way of interpreting this situation. The neighboring Mg atoms and the centers of the Cu tetrahedra together trace a cube. Indeed, if we replace all of the Cu tetrahedra with atoms at their centers (requiring perhaps an atom with a larger atomic radius), a BCC/Heusler framework is obtained. Such a substitution scheme has, in fact, been used to explain the Laves/Zintl intergrowth structures of K_3_Au_5_In, K_3_Au_5_Tl, and Rb_2_Au_3_Tl in terms of a parent Laves phase structure.^80^

**Figure 6:**
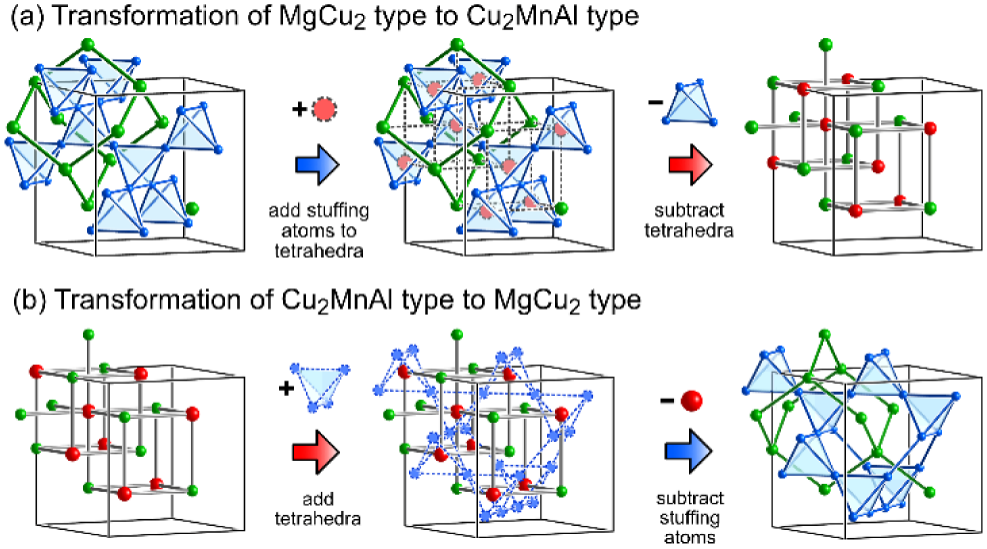
Scheme for the interconversion of the MgCu_2_-type Laves phase structure and the Heusler (Cu_2_MnAl-type) structure: (a) replacing the MgCu_2_-type vertex-sharing tetrahedra with atoms at the center yields a BCC-like framework characteristic of the Heusler structure; (b) the reverse process is achieved by replacing the atoms of one diamond network within the Heusler structure with atoms at their midpoints, creating vertex-sharing tetrahedra.

One can, of course, go the other way as well (Figure 6b). As illustrated so vividly by the Zintl phase NaTl,^81^ the BCC structure can be decomposed into two interpenetrating diamond networks. If we take one of these networks and place atoms at the edge centers, then a network of vertex-sharing tetrahedra emerges. Removal of the original atoms at the centers of the new tetrahedra yields the MgCu_2_ type. The applicability of this scheme to PrMg_1.6_Zn_5.4_ is illustrated by the Zn18 site’s environment (Figure 3a, lower left), where the near presence of a full Mg_4_Pr_4_ cube is interrupted by the replacement of a Pr atom with a Zn tetrahedron that directs a triangle of Zn12 atoms toward Zn18. This pattern, down to the orientation of the tetrahedron, closely resembles the process of Figure 6b applied to a single atom. Similarly, the coordination environment of the Pr1 site (Figure 4) can be directly derived from Figure 6b if we retain one Pr atom while replacing the others. The continued presence of the Pr atom reduces the newly formed tetrahedra to triangles, creating an alternation of atoms and triangles in a cube-like arrangement, mirroring the surroundings of the Pr1 site.

### 3.6 Interpretation of the Structure in Terms of Chemical Pressure Relief

So far, we have focused on making sense of the complex structure adopted by PrMg_1.6_Zn_5.4_. Beginning with the Mg sites and working outward, we have seen that this complexity can be reduced to two basic components: a Heusler-type matrix and Laves phase inclusions. The recognition of this theme puts us in a position to explore the driving forces that lead to the formation of this host–guest arrangement. We simply consider in what ways the PrMg_1.6_Zn_5.4_ structure offers energetic advantages over separate Heusler and Laves phases.

Some considerations regarding the presence or absence of related compounds in the system provide clues to this question. The feasibility of using Laves phase-type units in the system is evident in the existence of MgZn_2_ along the Mg–Zn binary edge of the Pr–Mg–Zn system. In addition, when Pr and Mg in PrMg_1.6_Zn_5.4_ are grouped in a compositional formula, we obtain (Pr/Mg)_2.6_Zn_5.4_ or (Pr/Mg)­Zn_2.08_. This ratio is quite close to the 1:2 ratio for MgZn_2_. However, the Pr–Mg–Zn phase diagram indicates no solubility of Pr in MgZn_2_ itself. This observation is consistent with the site preferences in the Laves phase blocks, with Mg and Zn atoms occupying sites corresponding to them in the parent structure and no noticeable Mg/Pr mixing. From this perspective, the complexity of PrMg_1.6_Zn_5.4_ could arise from the need to accommodate Pr in a way other than elemental substitution in the Laves phase.

An examination of the context for the Heusler domains leads to a different, complementary story. While the Pr–Mg–Zn system does contain a compound nominally related to the Heusler type, PrMg_3_,^82^ and it exhibits Zn solubility to reach the composition PrMg_1.4_Zn_1.6_, no refined crystal structure appears to be available for this ternary range. Instead, a clearer reference point is the ternary Heusler phase YMg_1.5_Zn_1.5_ in the related Y–Mg–Zn system, where no analogue to the complex PrMg_1.6_Zn_5.4_ has yet been characterized_,_ ^64,65^ Given the similar electronegativities of Y and Pr, and their common tendencies toward the formation of 3+ cations, it seems their substitution would create mainly perturbations due to the different spatial requirements of the two elements, with the larger size of Pr atoms compared to Y atoms leading to greater packing tensions. Here, the incorporation of MgZn_2_-type domains might then serve to relieve these packing issues, consistent with our observation that the Pr atoms in PrMg_1.6_Zn_5.4_ are all placed at boundaries between Heusler and Laves regions.

This hypothesis can be pursued theoretically using DFT-Chemical Pressure (CP) analysis, which uses the output of DFT calculations to construct representations of the interatomic pressures that arise within structures from packing issues. In Figure 7a, we present the resulting CP scheme for a model of the Y–Mg–Zn Heusler phase with the composition YMgZn_2_, where the Zn atoms form a primitive cubic network, with the Mg and Y atoms lying in alternating cubic holes to create a NaCl-type arrangement. Here, we have selected a group of atoms to match the environment of the Mg3 site in PrMg_1.6_Zn_5.4_. The pressures experienced by the atoms are presented through radial plots, where the distance between an atom and its corresponding surface is proportional to the sum of the pressure contributions along that direction. The sign of the pressure is indicated by the color of the surface, with black corresponding to negative CP (overly sparse packing, distances too long) and white indicating positive CP (overly dense packing, distances too short).

**Figure 7:**
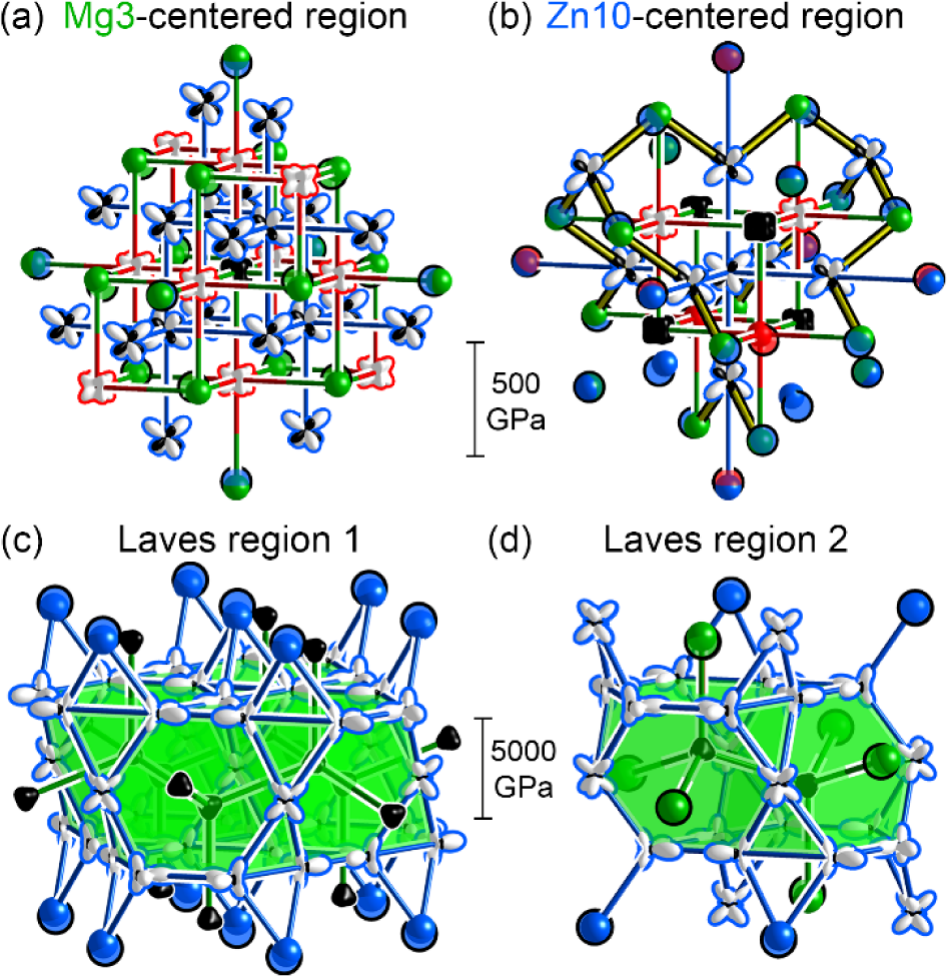
DFT-Chemical Pressure (CP) schemes of a YMgZn_2_ Heusler structure and the MgZn_2_ Laves phase, drawn in the contexts in which they arise in PrMg_1.6_Zn_5.4._ Fragments of the Heusler structure are drawn to match the surroundings of the (a) Mg3 and (b) Zn10 sites, while the fragments of MgZn_2_ are shown corresponding to Laves regions (c) 1 and (d) 2, corresponding to the Friauf polyhedra centered by the Mg5 and Mg6 sites, respectively. Spheres are drawn instead of CP lobes where *GrowDomain* finds a sizable mismatch in the atomic positions between the parent structure and its counterpart in PrMg_1.6_Zn_5.4_ or a change in the elemental identity (except where in PrMg_1.6_Zn_5.4_ mixed occupancy arises). Solid spheres represent the parent structure positions, while partially transparent spheres with black outlines show the positions in PrMg_1.6_Zn_5.4_. Red: Pr. Green: Mg. Blue: Zn.

The Y atoms here exhibit positive CPs, suggestive of the atoms being too large for their coordination environments. They exhibit a cube-shaped distribution with the rounded corners pointing to the surrounding Zn atoms, indicating Y–Zn contacts that are overly close. The desired expansion of the structure, however, is prevented by negative CPs between the Zn and Mg atoms, the latter of which appear as black cubes, experiencing negative CP from all directions. Overall, the Y atoms, with their large size, are holding open a lattice that is too spacious for the Mg atoms. The replacement of Y with Pr would intensify this effect.

With the ability of the *GrowDomain* program to effect an overlay between structures with analogous motifs, we can examine how these features relate to the positions of atoms surrounding this unit in PrMg_1.6_Zn_5.4_. The deviations from an idealized Heusler structure in PrMg_1.6_Zn_5.4_ are shown on the outskirts of the unit in Figure 7a for atoms exhibiting a large displacement or change in elemental identity. In such cases, rather than plotting a CP surface, we use spheres, with solid ones showing the idealized position and site coloring, and partially transparent ones representing the atoms in PrMg_1.6_Zn_5.4_. In each case, the modified atom hails from a Mg position in the idealized Heusler parent structure and exhibits a displacement into the domain. For those atoms that remain Mg in the intergrowth structure, they are displaced along an Mg–Zn negative CP toward a Zn atom deeper in the domain. For the remaining atoms, they switch to Zn in the ternary phase but still move toward other Zn atoms with negative CP lobes pointing in their direction. These features hint that the intergrowth with the Laves units has given the Heusler domain freedom to relax some of its internal packing stresses.

This theme can also be gleaned from the corresponding image of a section of the Heusler domain centered on the Zn10 site (Figure 7b). Two Pr atoms around the Zn10 position have jumped outward from the cube along positive CPs, pushing Zn atoms on the opposite side further out as well. The remaining features, though, largely stem from Mg-to-Zn and Pr-to-Zn substitutions farther out. While these substitutions are harder to identify as mechanisms of CP relief, their displacements can be interpreted in this way: nascent Zn atoms move away from new Pr atoms toward negative CP lobes on Zn atoms originally part of the domain.

The features of the Laves phase units can be similarly analyzed. In Figure 7c, we show an overlay of the MgZn_2_ CP scheme on the larger Laves phase fragment (region 1). The packing tensions within MgZn_2_ are based on a competition between positive CPs within the Zn sublattice, which call for the expansion of the structure, and negative CPs along the Mg–Zn and Mg–Mg contacts. PrMg_1.6_Zn_5.4_ in fact, has layers of Zn atoms above and below the sheet of Friauf polyhedra that continue the MgZn_2_-type connectivity; they are displaced outward, in line with the positive Zn–Zn CPs. These effects are even clearer in the smaller Laves phase fragment (region 2, Figure 7d), where not only are some of the Zn atoms continuing the MgZn_2_ connectivity pushed outward, but the Mg atoms on the surface of the Friauf polyhedra are pulled inward along negative CPs.

Together, these correlations highlight the opportunities that Laves phase/Heusler intergrowth creates for the release of packing tensions within them and the responsiveness of the structure’s atomic positions to the CP issues of the parent structures. Perhaps more importantly, though, they validate the picture of (1) the Pr atoms being overly compressed in the Heusler phase and (2) the preference for expanded coordination polyhedra driving the incorporation of the Laves phase units into the Heusler matrix.

## 4 Conclusions

Motivated by our earlier theory-guided discovery of the modular structure of PrMg_4_Zn_10_, we set out here to explore how this theme extends to other compounds in the Pr–Mg–Zn system. The anticipation of further complex, modular arrangements has been confirmed in our synthesis and structure determination of PrMg_1.6_Zn_5.4_, in which a matrix based on the Heusler structure serves as a host to inclusions of a Laves phase to create a giant cubic unit cell. The merging of the parent structures is facilitated by a simple geometric relationship between them, in which the BCC framework of the Heusler structure and the MgCu_2_-type Laves phase are interchanged by the substitution of single atoms with vertex-sharing tetrahedra. PrMg_1.6_Zn_5.4_ represents an intermediate stage in this process, in which the domain interfaces provide relatively open spaces for the Pr atoms. The formation of these new sites for the Pr atoms appears to drive the structure’s complexity, as confirmed by the positive CPs they would experience in a simple Heusler compound and their lack of solubility in the MgZn_2_ Laves phase.

In this analysis, the role of CP again comes to the foreground in the origin of complex intermetallic structures. However, the mechanism of its structural influence differs from our expectations. We originally approached the system with the hypothesis that CP relief at interface nuclei, motifs shared by two parent structures at an interface between them, would guide the formation of modular arrangements in the Pr–Mg–Zn system. Such was the case in PrMg_4_Zn_10_,^24^ and we have, in fact, applied this approach, in the form of matching rules for Frank-Kasper polyhedra, to explain the giant cubic unit cell adopted by Samson’s classic compound *β-*Al_3_Mg_2_.^83^ In PrMg_1.6_Zn_5.4_, continuity in the atomic positions across the parent structure domains is indeed evident, but it is difficult to identify distinct 3D units that are shared between the Heusler and Laves phase structures. Instead, the interfaces provide an opportunity to accommodate atoms that do not fit well into either of the parent structures individually. The resulting arrangement resembles a segregation of impurity elements to a grain boundary.^57^ It would be interesting to see if similar effects may underlie the complex 480-atom unit cell of Ce_20_Mg_19_Zn_81_ which bears similarities to PrMg_1.6_Zn_5.4_ in composition (but has less well-developed Laves phase and BCC units),^84^ as well as the observation of distinct elemental site preferences within different shells of the building blocks of the Tb_117_Fe_52_Ge_112_-type structures.^17^

The unexpected encounter with a new mechanism for structural complexity brings the role of design principles, such as the interface nucleus approach, to the foreground. Our pursuit of new structures in the Pr–Mg–Zn system was based on the assessment that ternary modular structures at interface nuclei would offer advantages in terms of atomic packing over real or plausible binary compounds within the system. The structure of PrMg_4_Zn_10_ confirms this point, but nothing in that statement precludes the possibility that other opportunities for new bonding may be even more favorable. In this way, the application of design principles to exploratory synthesis helps reveal and chart the competition among the increasing number of ways intermetallic phases are observed to incorporate complexity to solve bonding challenges. As we continue in this endeavor, we look forward to seeing what other forms of structural chemistry may surpass our designs.

## Supplementary Material



## Notes

Tables of refined atomic coordinates, atomic displacement parameters, and interatomic distances; SEM-BSE images and WDS data points; magnetic properties data; optimized cell parameters and atomic positions for a Heusler structure with the composition YMgZn_2_ (PDF)
