# Supplementary material for: The 2,240-Atom Unit Cell of PrMg1.6Zn5.4: An Intergrowth of the Laves and Heusler Structures Illustrating a Mechanism for the Stabilization of Complex Intermetallics
Source: J Am Chem Soc. 2025 Sep 16;147(39):35818–28. doi: 10.1021/jacs.5c12386 (PMC12503357; doi:10.1021/jacs.5c12386)
Supplement: Supplementary file 1 [file ja5c12386_si_001.pdf]

Supporting Information

The 2,240-Atom Unit Cell of $PrMg_{1.6}Zn_{5.4}$: An Intergrowth of the Laves and Heusler Structures Illustrating a Mechanism for the Stabilization of Complex Intermetallics

Danica G. Gressel, Leah C. Garman, and Daniel C. Fredrickson*

Department of Chemistry, University of Wisconsin-Madison,

1101 University Avenue, Madison, Wisconsin 53706, United States.

* Corresponding Author, e-mail: danny@chem.wisc.edu

S1. Crystallographic Data Tables for $PrMg_{1.6}Zn_{5.4}$

Table S1. Refined Atomic Coordinates for $PrMg_{1.6}Zn_{5.4}$.

Site	Wyckoff	*x*	*y*	*z*	U_{equiv}, (Å^2)	Occupancy
Pr1	8*a*	0	0	0	0.00938(18)	1
Pr2	48*f*	0.389668(18)	0	0	0.01215(14)	1
Pr3	96*g*	0.194989(8)	0.194989(8)	0.998943(12)	0.00921(10)	1
Pr4	32*e*	0.395521(12)	0.604479(12)	0.604479(12)	0.00953(10)	1
Pr5	96*g*	0.301057(8	0.604707(13)	0.698943(8)	0.00875(10)	1
Zn1	48*f*	0.29735(4)	1/2	1/2	0.0122(3)	1
Zn2	96*g*	0.23325(3)	0.678090(19)	0.678090(19)	0.0135(2)	1
Zn3	32*e*	0.30352(3)	0.69648(3)	0.69648(3)	0.0115(2)	1
Zn4	32*e*	0.34817(3)	0.65183(3)	0.65183(3)	0.00923(17)	1
Zn5	48*f*	1/4	0.65734(4)	3/4	0.0108(3)	1
Zn6	192*i*	0.249240(19)	0.647221(19)	0.55298(2)	0.0114(2)	1
Zn7	96*h*	0.267740(19)	5/8	0.482260(19)	0.0116(2)	1
Zn8	96*g*	0.34307(3)	0.553460(19)	0.553460(19)	0.0118(2)	1
Zn9	96*h*	0.178383(18)	5/8	0.571617(18)	0.0114(2)	1
Zn10	48*f*	1/4	3/4	0.55006(4)	0.0162(3)	1
Zn11	96*g*	0.30382(3)	0.607639(19)	0.607639(19)	0.0116(2)	1
Zn12	96*g*	0.37440(3)	0.570042(19)	0.429958(19)	0.0143(2)	1
Zn13	96*g*	0.23369(3)	0.626042(19)	0.626042(19)	0.0138(2)	1
Zn14	192*i*	0.322018(19)	0.625943(19)	0.53760(2)	0.0150(2)	1
Zn15	16*d*	3/8	5/8	3/8	0.0130(3)	1
Zn16	192*i*	0.320465(18)	0.571442(18)	0.482882(19)	0.0124(2)	1
Zn17	32*e*	0.45099(4)	0.54901(4)	0.54901(4)	0.0171(4)	0.807(9)
Zn18	32*e*	0.45001(4)	0.54999(4ss)	0.45001(4)	0.0143(5)	0.505(9)
Mg1	96*g*	0.39988(6)	0.60012(6)	0.50653(8)	0.0131(7)	1
Mg2	96*g*	0.29725(6)	0.70275(6)	0.50624(8)	0.0108(6)	1
Mg3	8*b*	1/2	1/2	1/2	0.0349(18)	1
Mg4	96*g*	0.29712(6)	0.79712(6)	0.59996(8)	0.0117(6)	1
Mg5	96*g*	0.65171(6)	0.15171(6)	0.04550(8)	0.0133(7)	1
Mg6	32*e*	0.09862(9)	0.09862(9)	0.09862(9)	0.0132(6)	1
Mg7	32*e*	0.45099(4)	0.54901(4)	0.54901(4)	0.0171(4)	0.193(9)
Mg8	32*e*	0.45001(4)	0.54999(4)	0.45001(4)	0.0138(5)	0.495(9)

Table S2. Refined Atomic Displacement Parameters for $PrMg_{1.6}Zn_{5.4}$.

Site	U_{11}	U_{22}	U_{33}	U_{12}	U_{13}	U_{23}
Pr1	0.0094(3)	0.0094(3)	0.0094(3)	0	0	0
Pr2	0.0146(3)	0.0109(2)	0.0109(2)	0	0	-0.0011(2)
Pr3	0.00912(15)	0.00912(15)	0.0094(2)	-0.00011(16)	-0.00011(11)	-0.00011(11)
Pr4	0.00953(17)	0.00953(17)	0.00953(17)	0.00052(16)	0.00052(16)	-0.00052(16)
Pr5	0.00869(14)	0.0089(2)	0.00869(14)	-0.00057(11)	0.00065(17)	0.00057(11)
Zn1	0.0142(7)	0.0112(4)	0.0112(4)	0	0	0.0014(5)
Zn2	0.0147(5)	0.0129(3)	0.0129(3)	-0.0007(3)	-0.0007(3)	0.0004(4)
Zn3	0.0115(3)	0.0115(3)	0.0115(3)	0.0015(4)	0.0015(4)	-0.0015(4)
Zn4	0.0092(3)	0.0092(3)	0.0092(3)	-0.0001(4)	-0.0001(4)	0.0001(4)
Zn5	0.0102(4)	0.0120(7)	0.0102(4)	0	-0.0001(5)	0
Zn6	0.0109(4)	0.0126(3)	0.0108(3)	-0.0002(3)	0.0006(3)	-0.0006(2)
Zn7	0.0110(3)	0.0126(5)	0.0110(3)	0.0006(3)	-0.0009(4)	0.0006(3)
Zn8	0.0127(5)	0.0114(3)	0.0114(3)	-0.0004(3)	-0.0004(3)	0.0010(4)
Zn9	0.0107(3)	0.0128(5)	0.0107(3)	0.0003(3)	-0.0010(4)	0.0003(3)
Zn10	0.0169(4)	0.0169(4)	0.0150(7)	0.0029(6)	0	0
Zn11	0.0115(5)	0.0117(3)	0.0117(3)	-0.0012(3)	-0.0012(3)	-0.0013(4)
Zn12	0.0138(5)	0.0145(3)	0.0145(3)	-0.0018(3)	0.0018(3)	0.0001(4)
Zn13	0.0139(5)	0.0137(3)	0.0137(3)	0.0005(3)	0.0005(3)	-0.0003(4)
Zn14	0.0150(4)	0.0144(4)	0.0156(4)	-0.0001(3)	-0.0002(3)	0.0002(3)
Zn15	0.0130(5)	0.0130(5)	0.0130(5)	0.0011(6)	-0.0011(6)	0.0011(6)
Zn16	0.0134(4)	0.0119(3)	0.0120(3)	0.0009(3)	0.0005(3)	-0.0015(3)
Zn17	0.0171(6)	0.0171(6)	0.0171(6)	-0.0024(5)	-0.0024(5)	0.0024(5)
Zn18	0.0138(8)	0.0138(8)	0.0138(8)	-0.0012(6)	0.0012(6)	-0.0012(6)
Mg1	0.0140(9)	0.0140(9)	0.0114(15)	-0.0001(11)	0.0002(8)	-0.0002(8)
Mg2	0.0115(8)	0.0115(8)	0.0095(13)	0.0010(11)	0.0004(8)	-0.0004(8)
Mg3	0.035(3)	0.035(3)	0.035(3)	0	0	0
Mg4	0.0118(9)	0.0118(9)	0.0114(14)	0.0008(12)	-0.0003(8)	-0.0003(8)
Mg5	0.0116(9)	0.0116(9)	0.0166(15)	-0.0007(11)	0.0016(8)	0.0016(8)
Mg6	0.0132(10)	0.0132(10)	0.0132(10)	0.0020(12)	0.0020(12)	0.0020(12)
Mg7	0.0171(6)	0.0171(6)	0.0171(6)	-0.0024(5)	-0.0024(5)	0.0024(5)
Mg8	0.0138(8)	0.0138(8)	0.0138(8)	-0.0012(6)	0.0012(6)	-0.0012(6)

Table S3. Selected Interatomic Distances for $PrMg_{1.6}Zn_{5.4}$.

Site	Neighbor	Distance (Å)	Size	Neighbor	Distance (Å)	Size	Neighbor	Distance (Å)
Pr1	Zn2 (×12)	3.5554(7)	Zn6	Pr5	3.0924(8)	Zn15	Zn12 (×6)	2.6812(7)
	Zn3 (×4)	3.1975(9)		Zn6	2.9732(9)		Mg5 (×6)	3.036(3)
	Zn5 (×6)	3.1964(14)		Zn7	2.6356(9)	Zn16	Pr2	3.4816(8)
Pr2	Zn1	3.1847(15)		Zn9	2.6412(9)		Pr3	3.4993(7)
	Zn8 (×2)	3.0636(8)		Zn10	3.5470(7)		Zn1	2.6568(8)
	Zn12 (×2)	3.4574(7)		Zn11	2.9941(10)		Zn7	2.5927(8)
	Zn16 (×4)	3.4816(8)		Zn13	2.6785(10)		Zn8	2.6307(10)
	Zn17 (×2)	3.1924(13)		Zn14	2.6690(9)		Zn12	2.6072(10)
	Zn18 (×2)	3.2064(15)		Mg2	3.002(2)		Zn14	2.6646(9)
	Mg1 (×4)	3.479(2)		Mg4	2.978(2)		Zn16	2.6502(9)
	Mg3	3.8061(7)	Zn7	Pr3 (×2)	3.1132(8)		Mg1	3.025(2)
	Mg7 (×2)	3.1924(13)		Zn6 (×2)	2.6356(9)		Mg2	2.963(3)
	Mg8 (×2)	3.2064(15)		Zn14 (×2)	2.6741(9)		Mg5	3.090(2)
Pr3	Zn1	3.1229(8)		Zn16 (×2)	2.5927(8)		Mg5	3.073(2)
	Zn6 (×2)	3.1132(8)		Mg2 (×2)	2.986(2)	Zn17/Mg7	Pr2 (×3)	3.1924(13)
	Zn6 (×2)	3.1823(7)		Mg5 (×2)	3.080(2)		Pr4	3.3144(13)
	Zn7 (×2)	3.5297(6)	Zn8	Pr2	3.0636(8)		Zn8 (×3)	3.7293(15)
	Zn8	3.1751(10)		Pr3	3.1751(10)		Zn18 (×3)	3.4154(19)
	Zn9 (×2)	3.5274(6)		Pr4	3.0771(9)		Mg1 (×3)	2.892(3)
	Zn10	3.2112(8)		Zn1	3.0479(10)		Mg3	2.9282(12)
	Zn11	3.1472(8)		Zn11	2.9698(10)		Mg8 (×3)	3.4154(19)
	Zn13	3.5167(7)		Zn14 (×2)	2.6606(10)	Zn18/Mg8	Pr2 (×3)	3.2064(15)
	Zn14 (×2)	3.5129(8)		Zn16 (×2)	2.6307(10)		Zn12 (×3)	2.7855(17)
	Zn16 (×2)	3.4993(7)		Mg1 (×2)	3.009(2)		Zn17 (×3)	3.4154(19)
	Mg2 (×2)	3.5469(19)		Mg7	3.7293(15)		Mg1 (×3)	3.128(3)
	Mg4	3.506(3)	Zn9	Pr3 (×2)	3.5274(6)		Mg3	2.9869(15)
Pr4	Zn4	2.8293(10)		Zn6 (×2)	2.6412(9)		Mg7 (×3)	3.4154(19)
	Zn8 (×3)	3.0771(9)		Zn9 (×2)	2.6043(6)	Mg1	Pr2 (×2)	3.479(2)
	Zn11 (×3)	3.1671(10)		Zn13 (×2)	2.6770(10)		Pr4	3.386(3)
	Zn14 (×6)	3.5072(8)		Mg4 (×2)	2.981(2)		Zn8 (×3)	3.009(2)
	Zn17	3.3144(13)		Mg6 (×2)	3.044(3)		Zn12 (×2)	2.971(3)
	Mg1 (×3)	3.386(3)	Zn10	Pr3 (×2)	3.2112(8)		Zn14 (×2)	3.026(2)
	Mg7	3.3144(13)		Pr5 (×2)	3.1238(9)		Zn16 (×2)	3.025(2)
Pr5	Zn2 (×2)	3.5209(9)		Zn1	3.3603(19)		Zn17	2.892(3)
	Zn3	3.1683(10)		Zn5	3.7008(19)		Zn18	3.128(3)
	Zn4	2.8152(10)		Zn6 (×4)	3.5470(7)		Mg5	3.091(3)
	Zn5	3.0823(9)		Mg2 (×2)	2.757(2)		Mg7	2.892(3)

	Zn6 (×2)	3.0924(8)
	Zn10	3.1238(9)
	Zn11 (×2)	3.1527(7)
	Zn13 (×2)	3.5024(8)
	Zn14 (×2)	3.4963(8)
	Mg2	3.402(3)
	Mg4 (×2)	3.393(2)
Zn1	Pr2	3.1847(15)
	Pr3 (×2)	3.1229(8)
	Zn8 (×2)	3.0479(10)
	Zn10	3.3603(19)
	Zn16 (×4)	2.6568(8)
	Mg2 (×2)	2.955(2)
Zn2	Pr1	3.554(7)
	Pr5 (×2)	3.5209(9)
	Zn2 (×2)	2.6911(12)
	Zn3	2.5847(13)
	Zn5 (×2)	2.6457(8)
	Zn13	2.5392(9)
	Mg4 (×2)	3.016(3)
	Mg6	3.110(3)
Zn3	Pr1	3.1975(9)
	Pr5 (×3)	3.1683(10)
	Zn2 (×3)	2.5847(13)
	Zn4	2.6681(13)
	Zn5 (×3)	2.9393(11)
Zn4	Pr4	2.8293(11)
	Pr5 (×3)	2.8152(10)
	Zn3	2.6681(13)
	Zn11 (×3)	2.6436(12)
Zn5	Pr1	3.1964(14)
	Pr5 (×2)	3.0823(9)
	Zn2 (×4)	2.6457(8)
	Zn3 (×2)	2.9393(11)
	Zn10	3.7008(19)
	Mg4 (×2)	3.034(2)
Zn6	Pr3	3.1132(8)
	Pr3	3.1823(7)
	Mg4 (×2)	2.872(2)
Zn11	Pr3	3.1472(8)
	Pr4	3.1672(10)
	Pr5 (×2)	3.1527(7)
	Zn4	2.6436(12)
	Zn6 (×2)	2.9941(10)
	Zn8	2.9698(10)
	Zn13	2.5804(13)
	Zn14	2.5751(10)
Zn12	Pr2	3.4574(7)
	Zn12 (×2)	2.7103(11)
	Zn15	2.6812(7)
	Zn16 (×2)	2.6072(10)
	Zn18	2.7855(17)
	Mg1 (×2)	2.971(3)
	Mg5 (×2)	3.076(2)
	Mg5	3.051(3)
	Mg8	2.7855(17)
	Pr3	3.5167(7)
	Pr5	3.5024(8)
	Zn2	2.5392(9)
	Zn6	2.6785(10)
	Zn9	2.6770(10)
	Zn11	2.5804(13)
	Mg4 (×2)	2.994(2)
	Mg6	3.097(3)
Zn14	Pr3	3.5129(8)
	Pr4	3.5072(8)
	Pr5	3.4963(8)
	Zn6	2.6690(9)
	Zn7	2.6741(9)
	Zn8	2.6606(10)
	Zn11	2.5751(10)
	Zn14	2.5387(9)
	Zn16	2.6648(9)
	Mg1	3.026(2)
	Mg2	2.987(2)
	Mg5	3.135(3)
	Mg8	3.128(3)
Mg2	Pr3 (×2)	3.5469(19)
	Pr5	3.402(3)
	Zn1	2.955(2)
	Zn6 (×2)	3.002(2)
	Zn7 (×2)	2.986(2)
	Zn10	2.757(2)
	Zn14 (×2)	2.987(2)
	Zn16 (×2)	2.963(3)
	Mg5	3.064(3)
Mg3	Pr2 (×6)	3.8061(7)
	Zn17 (×4)	2.9282(12)
	Zn18 (×4)	2.9869(15)
	Mg7 (×4)	2.9282(12)
	Mg8 (×4)	2.9869(15)
Mg4	Pr3	3.506(3)
	Pr5 (×2)	3.393(2)
	Zn2 (×2)	3.016(3)
	Zn5	3.034(2)
	Zn6 (×2)	2.978(2)
	Zn9 (×2)	2.981(2)
	Zn10	2.872(2)
	Zn13 (×2)	2.994(2)
	Mg6	3.076(4)
Mg5	Zn7 (×2)	3.080(2)
	Zn12 (×3)	3.076(2)
	Zn14 (×2)	3.135(3)
	Zn15	3.036(3)
	Zn16 (×2)	3.090(2)
	Zn16 (×2)	3.073(2)
	Mg1	3.091(3)
	Mg2	3.064(3)
	Mg5 (×2)	3.167(3)
Mg6	Zn2 (×3)	3.110(3)
	Zn9 (×6)	3.044(3)
	Zn13 (×3)	3.097(3)
	Mg4 (×3)	3.076(4)
	Mg6	3.152(4)

S2. WDS Data for $PrMg_{1.6}Zn_{5.4}$

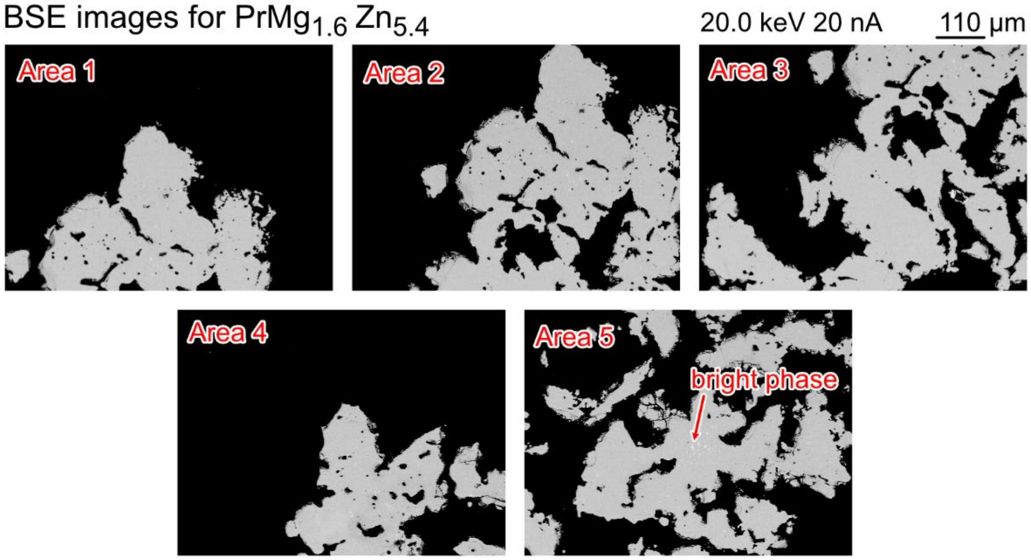

Figure S1. Scanning Electron Microscope-Back-Scattered Electron (SEM-BSE) image of polished sections of a sample containing $PrMg_{1.6}Zn_{5.4}$.

Table S4. WDS Compositions for Different Points on the Main Phase of the $PrMg_{1.6}Zn_{5.4}$ Sample.

Area	Line Number	Mg AT%	Pr AT%	Zn AT%	TOTAL
1	426	23.933	12.3416	63.7254	100
1	427	24.1878	12.3621	63.4502	100
1	428	23.8971	12.3138	63.7891	100
1	429	23.8164	12.3246	63.859	100
1	430	24.0556	12.3468	63.5976	100
1	431	23.8533	12.3053	63.8414	100
1	432	24.2582	12.3198	63.422	100
1	433	24.094	12.3853	63.5206	100
1	434	24.249	12.1938	63.5572	100
1	435	24.0139	12.2037	63.7824	100
2	436	24.0151	12.4366	63.5483	100
2	437	23.9253	12.3586	63.7161	100
2	438	23.8629	12.3584	63.7787	100
2	439	23.7737	12.2946	63.9317	100
2	440	23.9419	12.2629	63.7952	100
2	441	23.9235	12.2817	63.7948	100
2	442	23.8716	12.2812	63.8472	100
2	443	23.9975	12.3424	63.6601	100
2	444	24.1973	12.2887	63.514	100
2	445	24.199	12.2653	63.5357	100
3	446	24.1568	12.2852	63.5581	100

3	447	23.9988	12.3233	63.6778	100
3	448	23.9534	12.2943	63.7523	100
3	449	23.8684	12.2927	63.8389	100
3	450	24.0588	12.3865	63.5548	100
3	451	24.2169	12.2953	63.4878	100
3	452	24.0626	12.369	63.5684	100
3	453	24.0958	12.3225	63.5817	100
3	454	24.1431	12.2457	63.6113	100
3	455	24.046	12.3667	63.5873	100
4	456	24.1424	12.3309	63.5267	100
4	457	24.1167	12.3006	63.5827	100
4	458	24.1723	12.2516	63.5761	100
4	459	24.158	12.2792	63.5629	100
4	460	23.9531	12.3437	63.7032	100
4	461	24.0978	12.5078	63.3944	100
4	462	24.1875	12.2697	63.5428	100
4	463	24.261	12.3031	63.4359	100
4	464	24.2412	12.3366	63.4221	100
4	465	24.1504	12.2243	63.6253	100
5	466	23.9725	12.2729	63.7546	100
5	467	24.0541	12.3309	63.6149	100
5	468	24.1465	12.8057	63.0478	100
5	469	24.1502	12.331	63.5188	100
5	470	24.0605	12.3058	63.6337	100
5	471	24.1425	12.2545	63.603	100
5	472	24.0827	12.2583	63.6591	100
5	473	23.9313	12.2557	63.813	100
5	474	24.0455	12.2096	63.7449	100
5	475	24.5075	12.2234	63.2691	100
Average of 50 points		24.0648	12.3169	63.6183	
Standard Deviation		0.14098	0.09140	0.16348	

Table S5. WDS Compositions for Different Points of the Bright Phase in the $PrMg_{1.6}Zn_{5.4}$ Sample.

Area	Line Number	Mg AT%	Pr AT%	Zn AT%	TOTAL
5, bright phase	476	25.0921	15.8918	59.0162	100
5, bright phase	477	25.471	17.3277	57.2013	100
5, bright phase	478	26.6094	19.8698	53.5208	100
5, bright phase	479	26.9286	18.5545	54.5169	100
5, bright phase	480	27.3801	19.6541	52.9658	100
Average of 5 points		26.29624	18.25958	55.4442	
Standard Deviation		0.975151	1.665777	2.576257	

S3. Magnetism of in $PrMg_{1.6}Zn_{5.4}$.

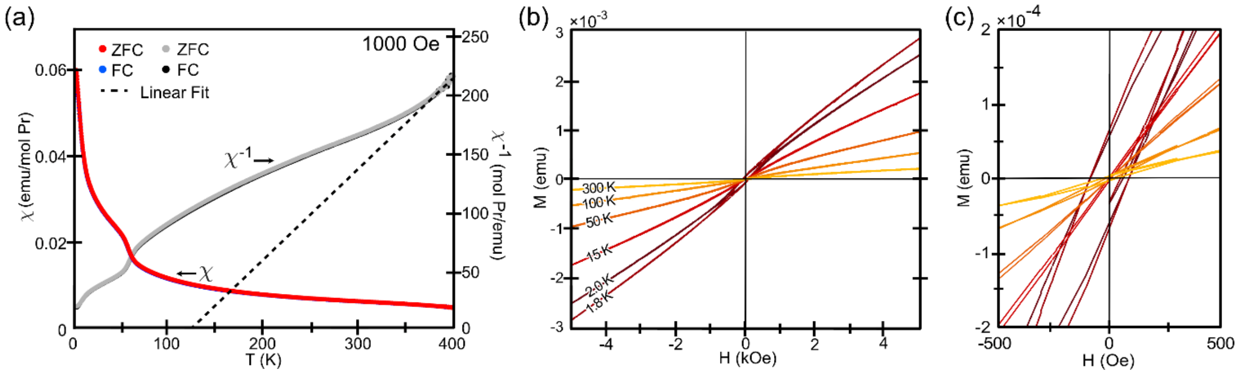

Figure S2. Magnetic data for $PrMg_{1.6}Zn_{5.4}$. (a) Temperature dependent ZFC/FC magnetic susceptibility curves measured in a 1,000 Oe field. The inverse susceptibility is plotted with gray (ZFC) and black (FC) circles. A black dotted linear trend line is drawn at the onset of the paramagnetic region, giving an approximate T_{cw} of 122 K. (b) Magnetization measured vs. magnetic field at various temperatures of 1.8 K, 2 K, 15 K, 50 K, 100 K and 300 K. A closer look at the magnetization curves (c) shows the hysteresis present at low temperatures.

S4. Additional Details Regarding Electronic Structure Calculations

Table S6. Unit Cell Vectors for the LDA-DFT-optimized Structure of $YMgZn_2$ in Cartesian Coordinates.

Formula	Structure type/total E	Vector	*x* (Å)	*y* (Å)	*z* (Å)
$YMgZn_2$	Cu_2MnAl	**a**	0	3.29241	3.29241
	-840.24257 eV/atom	**b**	3.29241	0	3.29241
		**c**	3.29241	3.29241	0

Table S7. LDA-DFT-optimized Fractional Atomic Coordinates for $YMgZn_2$.[a]

Element	*x*	*y*	*z*
Y	0	0	0
Mg	½	½	½
Zn	¼	¼	¼
Zn	¾	¾	¾

[a]All atoms are on high-symmetry positions, without degrees of freedom for optimization. The only free parameter for the structure is the *a* lattice parameter of the conventional face-centered cubic cell.
